# Recent Applications of Theoretical Calculations and Artificial Intelligence in Solid-State Electrolyte Research: A Review

**DOI:** 10.3390/nano15030225

**Published:** 2025-01-30

**Authors:** Mingwei Wu, Zheng Wei, Yan Zhao, Qiu He

**Affiliations:** 1 The College of Materials Science and Engineering, Sichuan University, Chengdu 610065, China; 2 International School of Materials Science and Engineering, Wuhan University of Technology, Wuhan 430070, China

**Keywords:** solid-state electrolytes, artificial intelligence, density functional theory, molecular dynamics, high-throughput screening

## Abstract

Solid-state electrolytes (SSEs), as key materials for all-solid-state batteries (ASSBs), face challenges such as low ionic conductivity and poor interfacial stability. With the rapid advancement of computational science and artificial intelligence (AI) technologies, theoretical calculations and AI methods are emerging as efficient and important virtual tools for predicting and screening high-performance SSEs. To further promote the development of the SSEs, this review outlines recent applications of theoretical calculations and AI in this field. First, the current applications of theoretical calculation methods, such as density functional theory (DFT) and molecular dynamics (MD), in material structure optimization, electronic property analysis, and ionic transport dynamics are introduced, along with an analysis of their limitations. Second, innovative applications of AI methods, including machine learning (ML) and deep learning (DL), in predicting material properties, analyzing structural features, and simulating interfacial behaviors are elaborated. Subsequently, the synergistic application strategies combining high-throughput screening (HTS), theoretical calculations, and AI methods are highlighted, demonstrating the unique advantages of integrating multiple methodologies in material discovery and performance optimization. Finally, the current research progress is summarized, and future development trends are forecasted. The deep integration of theoretical calculations and AI methods is expected to significantly accelerate the development of high-performance SSE materials, thereby driving the industrial application of ASSBs.

## 1. Introduction

Amidst escalating global warming challenges, electric vehicles (EVs) have emerged as pivotal solutions, leveraging clean energy utilization and effective exhaust emission reduction [1]. Nevertheless, the development of EVs grapples with various technical hurdles, notably limited driving range and safety concerns, attributed to the constraints of current commercial batteries [2]. Particularly in low-temperature environments, the substantial decline in battery performance directly impacts vehicle functionality. Moreover, the occurrence of battery safety incidents, such as thermal runaway leading to fires [3], presents a critically narrow rescue timeframe [4], accentuating the pressing need to fortify battery safety measures. Consequently, the advancement of the electric vehicle industry hinges on the development of battery technologies that seamlessly integrate high energy density with safety and stability [5].

The prevailing lithium-ion batteries with liquid electrolytes in commercial EVs exhibit commendable ionic conductivity but are hindered by flammability and toxicity, posing safety and reliability concerns [6]. In contrast, the emergence of solid-state electrolytes (SSEs) offers a promising avenue for future EV and large-scale energy storage device development, characterized by non-flammability and robust mechanical properties [7,8,9,10]. SSEs can be categorized into polymer, inorganic (oxides, sulfides, halides [11,12,13,14,15]), and organic–inorganic composite types [10,16,17,18,19,20,21]. Sulfide electrolytes, boasting superior room-temperature ionic conductivity exceeding 10 mS/cm, face challenges like moisture sensitivity and poor cathode material compatibility [22,23]. Strategies such as dopant optimization [24,25], interface modification [26,27,28,29], and 3D conductive network construction [30,31] have been developed to address these hurdles. However, industrial SSE application necessitates solutions to enhance ionic conductivity [32], mitigate interfacial side reactions [33,34,35], and resolve electrode volume-induced contact issues [30].

To address these obstacles, the fusion of theoretical calculations and artificial intelligence (AI) has opened novel avenues for designing and developing SSE materials. Density functional theory (DFT) calculations unveil intrinsic properties, band structures, and atomic-scale ion transport mechanisms, while molecular dynamics (MD) simulations accurately forecast ionic diffusion behavior and interfacial stability [36,37,38,39]. However, conventional theoretical methods are often computationally demanding and time-consuming, limiting their application in large-scale materials screening. In recent years, the integration of AI technologies, including machine learning (ML) and deep learning (DL), has markedly expedited the discovery of innovative SSEs. By establishing high-throughput computational platforms and training AI models with high-quality data from DFT/MD calculations, researchers can swiftly predict material properties and screen promising high-performance candidates [40,41,42]. This research strategy, harmonizing theoretical calculations with AI, not only significantly boosts materials screening efficiency but also offers distinctive advantages in predicting crucial performance metrics (e.g., ionic conductivity, electrochemical windows) and optimizing interfacial structure design. This approach furnishes robust theoretical backing for tackling fundamental scientific challenges in the SSE domain [43,44,45,46]. With the evolution of computational capabilities and algorithmic advancements, the deep integration of theoretical calculations and AI is poised to accelerate SSE development and innovation, propelling the industrialization of all-solid-state batteries (ASSBs).

In this study, we methodically investigate theoretical calculation techniques in SSE research, delineating their present uses and conducting a critical analysis of their constraints. We then clarify how AI technologies surmount these limitations, with a particular emphasis on the utilization of ML and DL in SSE research. Finally, we encapsulate the amalgamated applications of theoretical calculations, AI methodologies, and high-throughput screening (HTS), concluding with an examination of the primary challenges and future trajectories of this interdisciplinary methodology.

## 2. Theoretical Calculation Methods and Applications

### 2.1. Overview of Theoretical Calculation Methods

The rapid advancement of computational science in recent years has furnished robust theoretical tools for SSE materials research [47]. Traditional experimental approaches necessitate significant human and material resources for material design, synthesis, and characterization, often entailing prolonged cycles and high costs. Additionally, these methods frequently struggle to directly observe and comprehend material interaction mechanisms at the microscopic level. The integration of theoretical calculation methods has effectively mitigated these challenges, facilitating a profound understanding of material structures and performance characteristics at molecular and atomic scales. Furthermore, these methods enable the prediction and screening of material properties before experimental validation, thereby significantly enhancing research efficiency [48,49,50].

In SSE research, the predominant theoretical calculation methods encompass DFT and MD calculations [51,52,53,54,55]. These methods, while distinct, complement each other: DFT calculations, rooted in quantum mechanical principles, forecast material ground state properties and electronic structure features, while MD calculations simulate atomic motion laws, elucidating the dynamic evolution processes of materials. The amalgamation of these approaches furnishes comprehensive theoretical guidance for the design and refinement of SSE materials [56,57,58,59,60,61,62].

#### 2.1.1. Density Functional Theory

DFT stands as a first-principles computational approach grounded in quantum mechanics, originating from the seminal theorems by Hohenberg and Kohn in 1964 [63]. These theorems establish that the ground-state electron density uniquely governs all system properties under a given external potential, and that the ground-state energy, subject to particle number conservation, is a functional of electron density. The Kohn–Sham equations, proposed by Kohn and Sham in 1965 [64], further refine this framework by transforming complex many-body interactions into a problem of single electrons navigating an effective potential field, thus encapsulating many-body effects within the exchange-correlation functional. The solution of the Kohn–Sham equations via the self-consistent field method yields the system’s lowest energy state and corresponding electron density distribution.

DFT calculations encompass structural optimization, single-point energy calculations, and transition state calculations [65,66,67]. Structural optimization determines material stability by minimizing energy through atomic position adjustments; single-point energy calculations analyze electronic properties post-optimization, such as band structures and charge distributions; transition state calculations employ methods like Climbing Image Nudged Elastic Band (CI-NEB) to identify minimum energy pathways for reactions or ion migration [68,69]. Additionally, phonon spectrum calculations assess material dynamical stability and provide insights into the system’s temperature-dependent properties, such as thermal expansion and heat capacity. These calculations also enable the determination of the Gibbs free energy, which is essential for studying the thermodynamic properties of the system, including pressure effects. Molecular electrostatic potential analysis elucidates intermolecular interactions [70,71,72,73]. However, phonon spectrum calculations and thermodynamic property evaluations require significant computational resources, especially for complex systems. Modern computational methods, while efficient, are still resource-intensive, making them a challenge even with state-of-the-art computing systems. In this context, ML techniques offer the potential to significantly reduce the computational burden, accelerating these analyses and broadening their applicability in materials science.

DFT calculations predict crucial properties for SSE materials [47,74,75,76,77,78]. Thermodynamic stability is evaluated through formation and binding energies; electronic and conductivity characteristics are assessed via band structure and density of states (DOS) analysis; ionic conductivity performance is predicted by determining energy barriers through CI-NEB calculations. DFT calculations also unveil microscopic electrode/electrolyte interface interactions, including binding energies and charge transfer mechanisms. Redox potential calculations can be conducted to predict materials’ electrochemical voltage windows, guiding electrolyte material design and screening [50,79,80,81,82].

#### 2.1.2. Molecular Dynamics

MD serves as a computational simulation method tracking microscopic particle trajectories, elucidating atomic motion laws by solving Newton’s equations of motion. This technique unveils insights into the ionic conductivity, structural stability, and thermodynamic properties of SSEs [83,84]. Presently, MD encompasses classical molecular dynamics, ab initio molecular dynamics (AIMD), and deep learning molecular dynamics (DeepMD) [85]. Classical MD employs empirical force fields to model interatomic interactions, suitable for long-term simulations of large systems. AIMD utilizes quantum mechanical approaches for real-time updates of interatomic interactions, offering advantages in studying chemical bond rearrangements and complex interactions. DeepMD leverages neural networks to learn force field models from ab initio calculations, striking a balance between computational efficiency and accuracy.

For exploring practical SSE materials, selecting appropriate simulation conditions is crucial for accurately describing ionic conduction behavior. Simulations commonly utilize the canonical ensemble (NVT) and isothermal–isobaric ensemble (NPT) to closely mimic battery operating environments. By analyzing ion mean square displacement (MSD), ionic diffusion coefficients are determined, enabling the calculation of material ionic conductivity using the Nernst–Einstein relation [86,87]. Coupled with assessments of ionic migration probability distributions, visualization of ion conduction channels and migration mechanisms within the lattice offers critical insights into material ionic conductivity performance.

MD calculations excel in predicting material dynamic performance under real-world conditions. By scrutinizing ion trajectories, these simulations unveil microscopic mechanisms of ionic conduction, identifying preferential diffusion pathways, temperature-dependent diffusion coefficients, and ion transport behaviors. When integrated with other computational methods like DFT, MD enhances the understanding of SSE performance characteristics across various scales, furnishing comprehensive theoretical guidance for material design and optimization.

### 2.2. Overview of Theoretical Calculation Methods

#### 2.2.1. Geometric Optimization and Stability Evaluation

**Figure 1:**
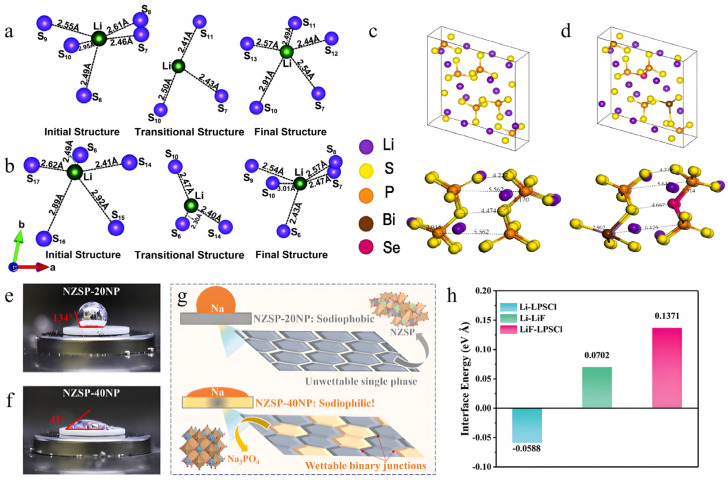
Diffusion of Li ion in (**a**) Li_7_P_3_S_11_ and (**b**) Li_7_P_3_S_10.25_O_0.75_. Reprinted with permission from ref. [70]. Copyright 2019, the Owner Societies. Structural model diagram and atomic spacing bond lengths of (**c**) LPS and (**d**) Bi_2_Se_3_-doped LPS. Reprinted with permission from ref. [88]. Copyright 2023, Elsevier B.V. Digital photographs of molten Na on (**e**) NZSP-20NP and (**f**) NZSP-40NP surfaces at 150 °C. (**g**) Schematic illustration of proposed wettability enhancement mechanisms for NZSP-40NP. Reprinted with permission from ref. [89]. Copyright 2024, Elsevier B.V. (**h**) Interface formation energy based on DFT calculation. Reprinted with permission from ref. [90]. Copyright 2024, Wiley-VCH GmbH.

In the theoretical investigation of SSE materials, structural characteristics represent the foundational and pivotal research focus. Computational analysis of materials’ geometric parameters and stability establishes a groundwork for comprehending performance mechanisms, offering crucial insights for material design and optimization. The analysis of bond lengths and angles in SSE materials through theoretical calculations unveils the strength of interactions between atoms and structural traits. Taking Li_7_P_3_S_11_ and Li_7_P_3_S_10.25_O_0.75_ [70] (Figure 1a,b) as examples, bond length assessments reveal relatively weak Li–S interactions, with bond lengths surpassing typical ionic bond lengths (1.13 Å). In Li_7_P_3_S_11_, Li–S bond lengths range from 2.46 to 2.95 Å, while in Li_7_P_3_S_10.25_O_0.75_, they range from 2.41 to 2.92 Å (Table 1). This subtle variance in bond lengths reflects the structural impact of oxygen doping on the structure and thus the ionic transport properties.

In the exploration of Bi_2_Se_3_-doped Li_2_S-P_2_S_5_ (LPS) SSEs [88] (Figure 1c,d), significant lattice parameter changes occur when Bi substitutes P and Se substitutes bridging S, influencing structural stability. Both experimental and theoretical calculations indicate that the a-axis expands from 6.21 Å to 6.42 Å, with comparatively minor alterations in the b-axis and c-axis. This anisotropic lattice modification directly impacts the dimensions and configuration of Li^+^ transport pathways, consequently enhancing the material’s ionic conductivity performance.

**Table 1:** Lattice parameters in LPS structures, Materials Project database results (“mp”) and optimized structures found in this study (“cal”) and optimized structures doped with Bi_2_Se_3_ (“cal”) [70].

Lengths (Å)	a	b	c
LPS (mp)	6.19	12.63	12.67
LPS (cal)	6.21	11.99	12.45
LPS-BiSe (cal)	6.42	12.05	12.51
**Angles (degree)**	**α**	**β**	**γ**
LPS (mp)	107.48	103.55	101.86
LiPS (cal)	107.72	102.52	101.85
LPS-BiSe (cal)	106.97	103.86	101.65

Mechanical properties play a crucial role in battery material design, particularly for SSEs. A recent study by Tanibata et al. demonstrated that shear modulus can serve as an effective predictive index for material deformability in all-solid-state lithium-ion batteries [91]. By computationally calculating shear moduli and experimentally evaluating six chloride compounds, the researchers established design guidelines: 1. Materials with shear modulus G < 12 GPa can achieve approximately 100% density through compaction; 2. Materials with shear modulus G < 18 GPa can minimize particle grain boundary resistance to around 10%. The study’s significance lies in providing a computational screening method for identifying materials with optimal mechanical properties, applicable not only to chloride materials but potentially extendable to oxide and sulfide battery materials. This approach offers a promising strategy for efficiently designing and selecting high-performance solid-state battery components by leveraging computational chemistry methods to predict mechanical characteristics.

Interfacial stability plays a vital role in SSE performance. The compatibility between SSEs and electrode materials is assessed through calculations of the work of adhesion (Wad) and binding energy. For instance, in the dual-phase Na_3_Zr_2_Si_2_PO_12_-Na_3_PO_4_ composite solid electrolyte [89] (Figure 1e–g), DFT calculations reveal superior affinity of Na_3_PO_4_ (NP) and its derivatives with metallic Na compared to Na_3_Zr_2_Si_2_PO_12_ (NZSP), validated by the wetting angle calculations. The wetting angle, computed using the Young–Dupré equation, aligns with experimental data, confirming the favorable interfacial wettability between NZSP-40NP and metallic Na. Moreover, in the investigation of anode interfaces within all-solid-state lithium metal batteries [90] (Figure 1h), the interface formation energy of Li-Li_6_PS_5_Cl (Li-LPSCl) stands at −0.0588 eV Å^−2^. Following the introduction of LiF, the interface formation energies of Li-LiF and LiF-LPSCl are 0.0702 eV Å^−2^ and 0.1371 eV Å^−2^, respectively. This outcome suggests that the incorporation of LiF substantially boosts interfacial stability, effectively curtailing the formation of lithium dendrites.

In SSE research, interfacial reaction calculations are a key strategy for addressing electrode–electrolyte compatibility challenges. Conventional solid-state batteries face severe interfacial degradation, primarily stemming from unfavorable chemical reactions between electrode materials and electrolytes. These side reactions result in rapid battery performance deterioration, significant capacity loss, and even complete failure. Tanibata et al. proposed an innovative approach to tackle this issue [92]. By applying the hard and soft acids and bases (HSAB) principle, combined with first-principles calculations and experimental validation, they revealed the interfacial chemical behaviors between different materials. The study demonstrated that traditional oxide electrodes, such as LiFePO_4_, exhibit severe acid-base reactions with LiAlCl_4_ electrolytes, leading to poor battery performance. Through precise screening, they identified Li_2_FeCl_4_ as an electrode material that successfully suppresses interfacial side reactions. This resulted in a fully solid-state lithium-ion battery with a reversible capacity of 100 mAh g^−1^ at 3.65 V. This computational approach not only significantly reduces experimental costs but also accelerates the identification of more stable material combinations.

#### 2.2.2. Electronic Structure Characteristics

Beyond geometric structural analysis, understanding the electronic structure characteristics of SSE materials is equally crucial for elucidating their electrochemical properties. Theoretical calculations, encompassing band structure, DOS distribution, and charge transfer analyses, offer profound insights into the electronic properties of materials and their correlation with ionic conduction mechanisms.

**Figure 2:**
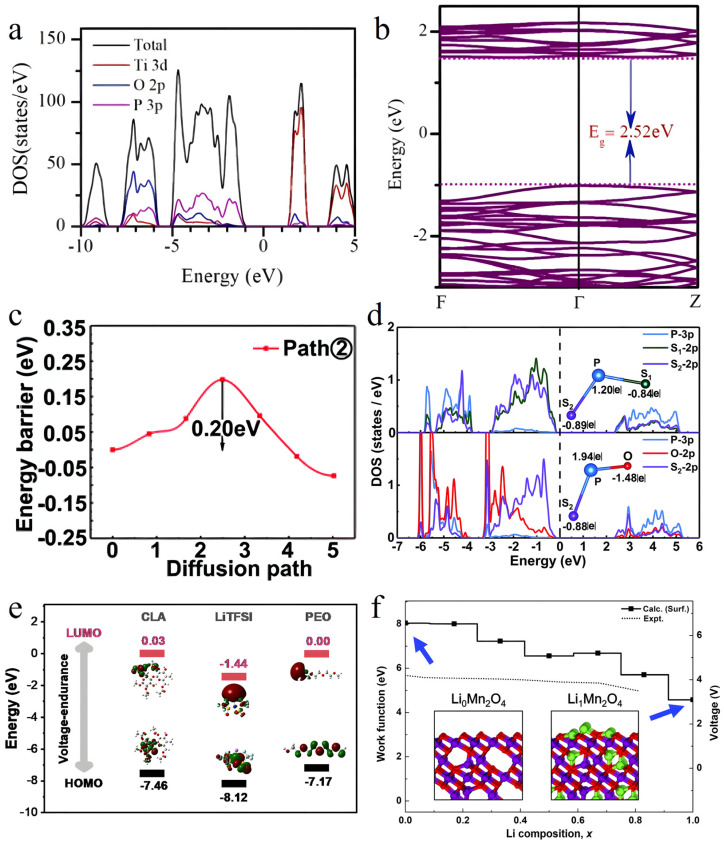
(**a**) Total and partial DOSs (PDOSs) of NaTi_2_(PO_4_)_3_. (**b**) Electronic band structure of NaTi_2_(PO_4_)_3_ along the high symmetry path F-Γ-Z. Reprinted with permission from ref. [93]. Copyright 2024, Elsevier Ltd. (**c**) The migration energy barriers of Li_7_P_3_S_10.25_O_0.75_. (**d**) The PDOSs and Bader charges for the S_1_–P–S_2_ and O–P–S_2_ clusters, respectively. Reprinted with permission from ref. [70]. Copyright 2019, the Owner Societies. (**e**) The HOMO and LUMO energy levels of the CLA and LiTFSI compared with common polymers. Reprinted with permission from ref. [94]. Copyright 2023, Wiley-VCH GmbH. (**f**) Work function and corresponding voltage (vs. Li^+^/Li) from LMO surface obtained from calculations (solid line with squares) and experiments (dotted line). The voltages were calculated with respect to the Li^+^/Li electrode. Reprinted with permission from ref. [95]. Copyright 2018, the Owner Societies.

Exploring the band structure of SSE materials, the NaTi_2_(PO_4_)_3_ (NTP) system serves as a representative case study [93]. DFT computations reveal that the valence band of NTP is predominantly constituted by oxygen 2p states, while the conduction band is chiefly influenced by titanium 3d states, showcasing a sizable bandgap of 2.52 eV (Figure 2a,b). This electronic structure characteristic signifies NTP’s role as an electronic insulator, with its limited electronic conductivity standing out as a significant trait of this SSE material.

Detailed analysis of the DOS offers comprehensive insights into the electronic structure of materials. For instance, in the Li_7_P_3_S_11−x_O_x_ series materials [70], variations in oxygen doping concentration leads to notable changes in electronic DOS distribution. Specifically, in Li_7_P_3_S_10.25_O_0.75_, the introduction of oxygen atoms not only impacts the distribution of electronic states at the valence band maximum but also alters the Li^+^ transport environment by regulating local electronic structure. Calculations indicate a reduced ionic migration barrier of 0.20 eV (Figure 2c,d). Furthermore, the analysis of molecular orbital energy levels and work function calculations provides crucial insights into studying electrode-electrolyte interfaces. In the investigation of cellulose acetate (CLA)-based polymer electrolytes [94], DFT computations reveal that the elevated lowest unoccupied molecular orbital (LUMO) energy level (−1.86 eV) of the CLA matrix signifies favorable compatibility with lithium metal anodes (Figure 2e).

Additionally, work function analysis serves to reflect electronic structure changes at interfaces. The work function, defined as the minimum internal energy required to promote an electron from the Fermi level to the vacuum level, is mathematically expressed as Φ = E_vac_ − E_F_. Byungchan Han’s team conducted a systematic investigation of the work function of the LiMn_2_O_4_ cathode surface using first-principles calculations [95]. The study employed a 3 × 3 × 1 k-point grid and incorporated the Hubbard U correction method (U = 3.9 eV) to ensure accurate calculations of the surface work function. By converting the theoretical work function into electrode potentials and benchmarking these against experimental measurements, the team demonstrated excellent agreement within the lithium-ion content range of x = 0–0.8 (Figure 2f). Moreover, the research unveiled a clear variation in the work function during proton transfer, offering microscopic insights into the electrochemical dynamics at the electrolyte–electrode interface. These comprehensive analyses of electronic structure characteristics establish a crucial theoretical foundation for understanding the fundamental properties of SSE materials. They underscore the indispensable role of theoretical calculations in unraveling the intricate electronic structures of materials.

#### 2.2.3. Ion Transport Kinetics

In the study of solid electrolyte materials, theoretical calculations serve as a crucial tool for elucidating ion migration mechanisms, particularly excelling in the analysis of ion hopping frequencies and diffusion dynamics. The ion migration process can be viewed as a series of random hopping events within the lattice, with rates typically following the Arrhenius relationship, closely linked to migration activation energy and temperature [96]. Theoretical calculations allow for the atomic-scale description of these migration processes, employing methods such as the Nudged Elastic Band (NEB) method, molecular dynamics (MD) simulations, bond valence (BV) models, and density functional theory (DFT).

The NEB method identifies the minimum energy path (MEP) between initial and final ion positions, providing key dynamic parameters such as migration paths and energy barriers. For instance, in Li_4_P_2_S_6_, NEB calculations revealed low-barrier migration paths, indicating excellent ion conduction potential [97]. In contrast, molecular dynamics simulations, particularly ab initio molecular dynamics (AIMD), capture additional dynamic information beyond migration paths by directly simulating ion trajectories and behaviors. This approach has uncovered a three-dimensional migration network within the planes of LGPS materials, offering crucial insights for optimizing material conductivity [98].

Furthermore, the BV model constructs three-dimensional energy distribution maps by analyzing the relationship between bond lengths and valences within the lattice, predicting ion migration paths and energy barrier distributions. In ZnO-doped Li_3_PS_4_, BV analysis demonstrated that doping optimizes migration paths and reduces local barriers, significantly enhancing ionic conductivity [99]. DFT reveals fundamental material properties from an electronic structure perspective and, when combined with NEB or AIMD, further quantifies ionic diffusion coefficients, migration barriers, and the thermodynamic stability of materials [100].

These methods also indicate that ion migration mechanisms primarily include vacancy, interstitial, and interstitial-vacancy coupling mechanisms, with specific mechanisms depending on the lattice characteristics and defect structures of the material [101,102]. Theoretical calculations not only explain the essence of ion hopping at the microscopic scale but also provide a solid theoretical foundation for optimizing the design of novel solid electrolyte materials. Through HTS and dynamic simulations, potential candidate materials with low barriers and high conductivity can be rapidly identified, paving the way for the development of next-generation high-performance electrolytes [103,104].

**Figure 3:**
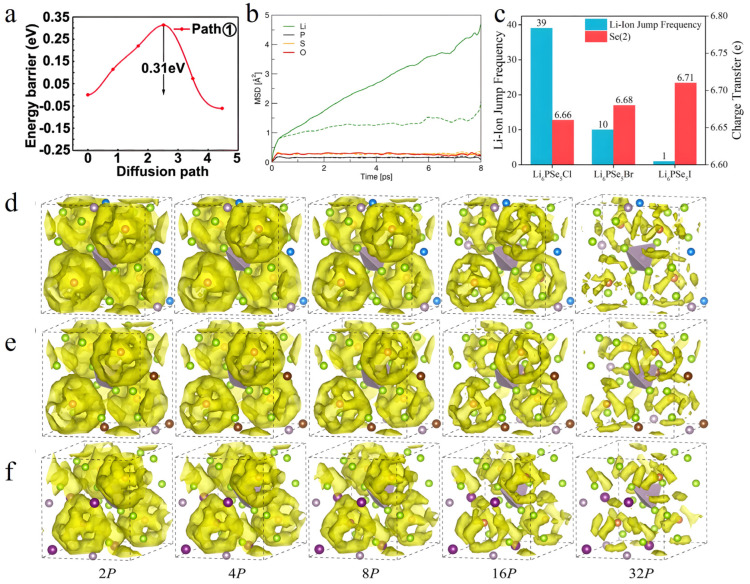
(**a**) The migration energy barriers of Li_7_P_3_S_11_. Reprinted with permission from ref. [70]. Copyright 2019, the Owner Societies. (**b**) The MSDs of Li, P, S and O atoms are presented by dashed lines for β-Li_3_PS_4_ and by solid lines for β-Li_3_PS_3.75_O_0.25_ [105]. (**c**) The total number of inter-cage jumps of Li ions in Li_6_PSe_5_X (X  =  Cl, Br, I) and the Bader charge transfer of Se(2) atoms located in the center of the migrating cages during the 50 ps AIMD simulation at 600 K. Caged Li-ion migration density plots for (**d**) Li_6_PSe_5_Cl, (**e**) Li_6_PSe_5_Br, and (**f**) Li_6_PSe_5_I at 600 K for iso-surface level 2*P*–32*P* (*P* was set at 2 × 10^−4^). To improve clarity, all individual Li atoms were hidden [106].

The assessment of ion migration paths offers a clear understanding of the transport mechanism. In the Li_7_P_3_S_11_ and Li_7_P_3_S_10.25_O_0.75_ systems [70], calculations utilizing the CI-NEB method unveiled that the migration barrier for Li ions in Li_7_P_3_S_11_ is 0.31 eV, whereas in O-doped Li_7_P_3_S_10.25_O_0.75_, the barrier decreases to 0.20 eV (Figure 2c and Figure 3a). This reduction in barrier is attributed to oxygen doping, which widens the diffusion path of Li^+^, effectively overcoming the bottleneck sites in the Li diffusion process.

MSD analysis can quantitatively depict ion diffusion behavior. Research indicates that in the undoped β-Li_3_PS_4_ system [105], the average migration distance of Li ions over 8000 simulation steps is approximately 1 Å, notably lower than the nearest-neighbor Li-Li distance (2.52 Å), suggesting that most Li ions oscillate around their equilibrium positions. Conversely, the MSD of Li ions in the O-doped system (β-Li_3_PS_3.75_O_0.25_) exhibits linear growth, indicating significantly enhanced ionic diffusion (Figure 3b).

Exploring the ionic migration probability distribution unveils preferential conduction pathways in materials. Using the Li_6_PSe_5_X (X = Cl, Br, I) system as a case study [106], AIMD simulations demonstrate that Li_6_PSe_5_Cl displays the lowest activation energy (0.21 eV) and the highest room-temperature Li^+^ conductivity (3.85 × 10^−2^ S cm^−1^). Analysis of Li^+^ migration characteristics within distinct cage-like channels reveals the constraining influence of Se(2) atoms at cage centers on Li^+^ migration, with cross-cage migration identified as pivotal for achieving three-dimensional conduction. Further, through ionic jump frequency analysis, it is confirmed that Li_6_PSe_5_Cl exhibits the weakest Se(2) binding to Li^+^, elucidating its superior ionic conductivity performance (Figure 3c–f).

These comprehensive analyses of dynamic features not only aid in comprehending the microscopic mechanisms of ionic conduction in SSEs but also offer crucial insights for the design of high-performance SSE materials.

### 2.3. Limitations and Challenges of Theoretical Calculation Methods

While theoretical calculation methods are pivotal in SSE research, they encounter several challenges in practical applications. One major challenge is the limitation of computational accuracy. In DFT calculations, the selection of exchange-correlation functionals directly impacts the accuracy of results. Commonly used local density approximation (LDA) and generalized gradient approximation (GGA) functionals tend to underestimate bandgap values for semiconductor and insulator materials. Although hybrid functionals like HSE06 offer improved accuracy with computational time increasing by approximately twice, this additional computational cost remains relatively reasonable for many research applications. Furthermore, beyond hybrid functionals, quasiparticle methods represent another advanced computational approach. These methods can achieve remarkable optical accuracy but come with exceptionally high computational requirements, particularly in terms of memory (random access memory) consumption. Moreover, computational models often rely on ideal crystal structures due to computational resource constraints, making it challenging to fully simulate defects, interfaces, and amorphous properties in materials. Most DFT calculations are conducted at 0 K, posing difficulties in accurately describing material behavior at operational temperatures. While AIMD simulations can incorporate temperature effects, their simulation durations are typically limited, hindering the representation of long-term dynamic behaviors in practical scenarios.

Discrepancies between theoretical calculations and experimental findings also pose a challenge. Ionic conductivities derived from MD calculations may deviate from experimental values, primarily due to computational models’ limitations in capturing the complex influences of defects, grain boundaries, and real-world processing techniques in materials.

### 2.4. Chapter Summary

This chapter systematically elucidates the utilization of theoretical calculation methods in SSE research. Beginning with the fundamental principles of DFT and MD calculations, it delves into the theoretical underpinnings of these approaches. In practical scenarios, theoretical calculations offer distinct advantages: they enable the prediction of material structural characteristics and thermodynamic stability through geometric structure optimization and stability analysis; provide profound insights into electronic properties and electrochemical performance via electronic structure characteristic analysis; and unveil the microscopic mechanisms of ionic migration in materials through the study of ionic transport dynamics.

Despite current challenges in computational accuracy and model simplification, advancements in computational technology are progressively addressing these issues. Notably, the rapid evolution of AI technology in recent years has introduced novel avenues for enhancing computational efficiency and reducing costs. By integrating AI methodologies with traditional theoretical calculation techniques, we can not only surmount existing limitations but also leverage available research data more effectively to bolster the advancement of innovative SSE materials. These innovative research approaches and tools are comprehensively discussed in the subsequent chapter.

## 3. Artificial Intelligence Methods and Applications

### 3.1. Overview of Artificial Intelligence Methods

Historically, SSE research has heavily relied on time-consuming and expensive experimental methods. While the integration of theoretical calculations has alleviated the inefficiencies of traditional experimental validation, computational challenges persist, especially in handling intricate material systems and extensive candidate material repositories. The advent of AI methodologies has ushered in a new era for SSE investigations. These data-centric approaches swiftly discern patterns from existing data, markedly enhancing the efficiency of materials screening [107,108,109].

AI methods can be broadly categorized into two groups based on their learning strategies: ML and DL. ML establishes relationships between material structure and properties using statistical algorithms, whereas DL employs multi-layer neural networks for intricate feature extraction and pattern recognition. These methodologies offer novel research avenues and tools for the design and advancement of SSE materials.

#### 3.1.1. Machine Learning Methods

ML methods can be classified into supervised learning, unsupervised learning, semi-supervised learning, and reinforcement learning based on differences in the learning process [110]. In the realm of SSE research, supervised learning and unsupervised learning are currently the most prevalent methodologies [111,112,113].

Supervised learning necessitates training with labeled data to establish mappings between input features and output results for predictive purposes [114]. Each sample in the training dataset comprises feature vectors and corresponding label values. Throughout training, the algorithm optimizes model parameters to minimize the error between predicted and actual values. Common supervised learning algorithms in SSE research, such as Support Vector Machine (SVM) [115], Logistic Regression (LogR) [116], and Decision Tree [117], are employed to establish quantitative relationships between material characteristics and performance, facilitating predictions of vital parameters like ionic conductivity and mechanical strength.

Unsupervised learning uncovers inherent patterns and structural features directly from unlabeled data. By analyzing similarities or distance relationships between data points, these methods automatically cluster or reduce sample dimensionality with akin characteristics, unveiling essential data patterns [110]. Cluster analysis, a prevalent unsupervised learning method in SSE research, aids in identifying new material types and patterns of property changes from extensive materials data [118,119].

Semi-supervised learning amalgamates the strengths of supervised and unsupervised learning by leveraging both labeled and unlabeled data for training, ideal for scenarios where obtaining labeled data is challenging or costly [120]. Reinforcement learning optimizes decision-making through iterative trials and evaluations, akin to a “trial and error” process, refining decisions by exploring various choices and learning from outcomes [121]. While these two methods are less commonly applied in SSE research presently, advancements in data volume and algorithmic enhancements may broaden their applications in the future [7].

#### 3.1.2. Deep Learning Methods

DL stands as a pivotal subset of ML, focusing on the automatic extraction and comprehension of intricate features through neural networks comprising multiple hidden layers [122]. Unlike conventional ML methods, DL obviates the need for manual feature crafting, instead autonomously deriving hierarchical feature representations from raw data.

In SSE research, Convolutional Neural Network (CNN) and Graph Neural Network (GNN) emerge as the predominant DL models. CNN is explicitly designed to handle data featuring regular grid structures. In the case of periodic crystalline materials, the atomic positions can be projected onto a voxel grid, effectively transforming them into a regular grid format that aligns well with CNN processing methodologies [123]. On the other hand, GNNs are adept at handling network structures representing interatomic interactions, enabling direct learning of material properties from atomic spatial configurations. Notably, the Crystal Graph Convolutional Neural Network (CGCNN), tailored for crystalline materials, has exhibited promising outcomes in forecasting properties of SSE materials [124].

The development of DL-driven potential functions represents a crucial research avenue. Unlike conventional molecular dynamics simulations that depend on empirical potentials or DFT calculations, DL-based potentials offer a substantial enhancement in computational efficiency without compromising quantum mechanical precision. By employing neural networks to model potential energy surfaces, this approach accurately characterizes interatomic interactions, a crucial aspect for investigating ion transport mechanisms within SSEs [85].

### 3.2. Applications of Artificial Intelligence in SSEs

#### 3.2.1. Performance Prediction and Screening of Materials

The advancement of high-performance SSE materials hinges on precise prediction and efficient screening of material properties. Leveraging extensive datasets, AI techniques can quickly forecast crucial material performance parameters, significantly enhancing screening efficacy. In SSE investigations, AI methodologies predominantly target ionic conductivity prognostication, stability assessment, and electrochemical performance prediction.

**Figure 4:**
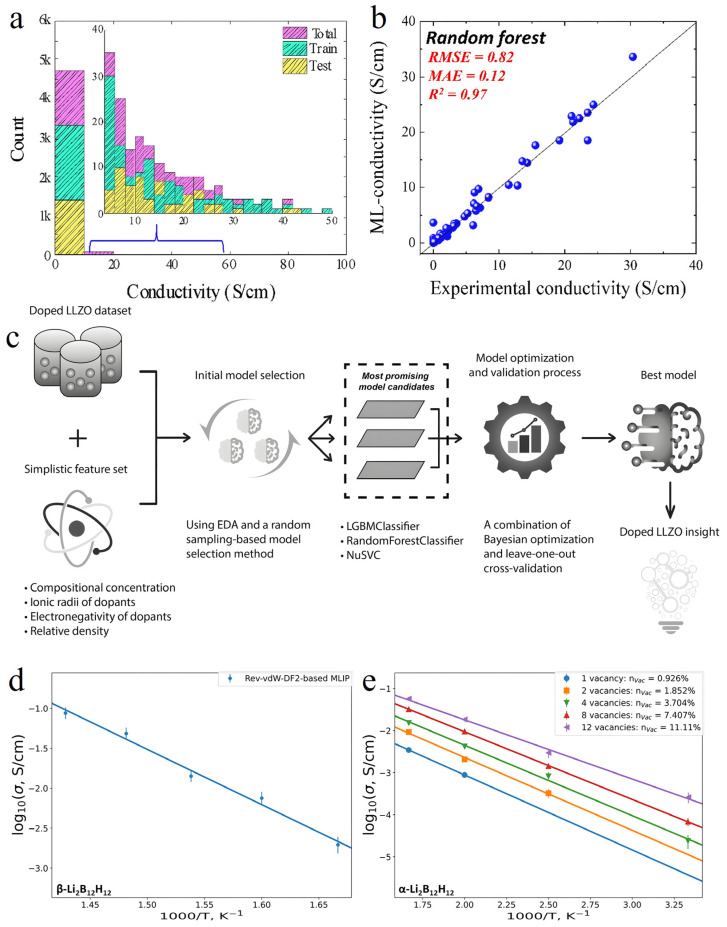
(**a**) Representative split used to show the testing results of the built model. (**b**) Testing results of the ML models built using RF. Reprinted with permission from ref. [125]. Copyright 2023, Elsevier B.V. (**c**) General approach to classification of doped LLZO ionic conductivity. Reprinted with permission from ref. [127]. Copyright 2021, Elsevier B.V. Ionic conductivity as a function of temperature calculated using the rev-vdW-DF2-based MLIP: (**d**) for the high-temperature β-Li_2_B_12_H_12_ without vacancies and (**e**) for α-Li_2_B_12_H_12_ with different concentrations of vacancies; vertical bars denote statistical errors; at temperatures above 400 K and high vacancy concentrations, the errors are small. Reprinted with permission from ref. [128]. Copyright 2023, American Chemical Society.

In terms of ionic conductivity prediction, Pereznieto et al. devised a forecasting model employing the Random Forest (RF) algorithm [125]. This model, constructed using 4826 ionic conductivity data points, identified 109 pivotal features for training. Demonstrating superior predictive accuracy (R^2^ = 0.97) compared to linear regression and AdaBoost algorithms, the RF model excelled in performance (Figure 4a,b). The team utilized this model to screen around 30,000 compounds sourced from the Inorganic Crystal Structure Database (ICSD), successfully pinpointing 67 promising Li–S compounds exhibiting high ionic conductivity (>0.01 S/cm). This study underscores the distinct advantages of ML in large-scale materials screening endeavors.

To predict SEE materials’ stability, Tang’s team employed the gradient descent-based XGBoost algorithm to systematically analyze the structural characteristics of inorganic SSEs [126]. Their study underscored the importance of SSEs possessing both low electronic conductivity and structural stability. By scrutinizing over 6000 structures, they identified 194 candidate materials meeting these dual criteria. This ML-driven screening approach markedly enhanced materials discovery efficiency.

For material performance optimization, Adhyatma et al. curated a dataset comprising 176 distinct samples for doped Li_7_La_3_Zr_2_O_12_ (LLZO) systems and utilized the Light Gradient Boosting Machine (LGBM) algorithm for performance prognostication [127] (Figure 4c). Leveraging Bayesian optimization and leave-one-out cross-validation, the model achieved a commendable prediction accuracy of 0.903. Notably, through feature importance analysis, they unearthed that the relative density of the electrolyte and the electronegativity of Li-site dopants significantly influenced the ionic conductivity of doped LLZO, offering clear optimization pathways for material design.

Furthermore, Maltsev et al.’s research introduced novel insights into material property prediction [128] (Figure 4d,e). Their developed Machine Learning Interatomic Potentials (MLIPs) method not only forecasts material structural stability but also accurately characterizes phase transition behavior across varying temperatures. Comparative analysis with experimental data revealed that MLIPs based on the rev-vdW-DF2 functional exhibited remarkable precision in predicting phonon DOSs, serving as a dependable tool for assessing materials’ thermodynamic stability.

These studies collectively highlight the substantial advantages of AI methodologies in performance prediction and SSE material screening. First, AI models efficiently handle extensive datasets, significantly enhancing screening efficacy. Second, through feature importance analysis, crucial factors influencing material performance are pinpointed, offering valuable insights for material design. Finally, the high accuracy and scalability of AI methods serve as a crucial link between theoretical calculations and experimental investigations.

#### 3.2.2. Structure Feature Analysis and Preparation Optimization

**Figure 5:**
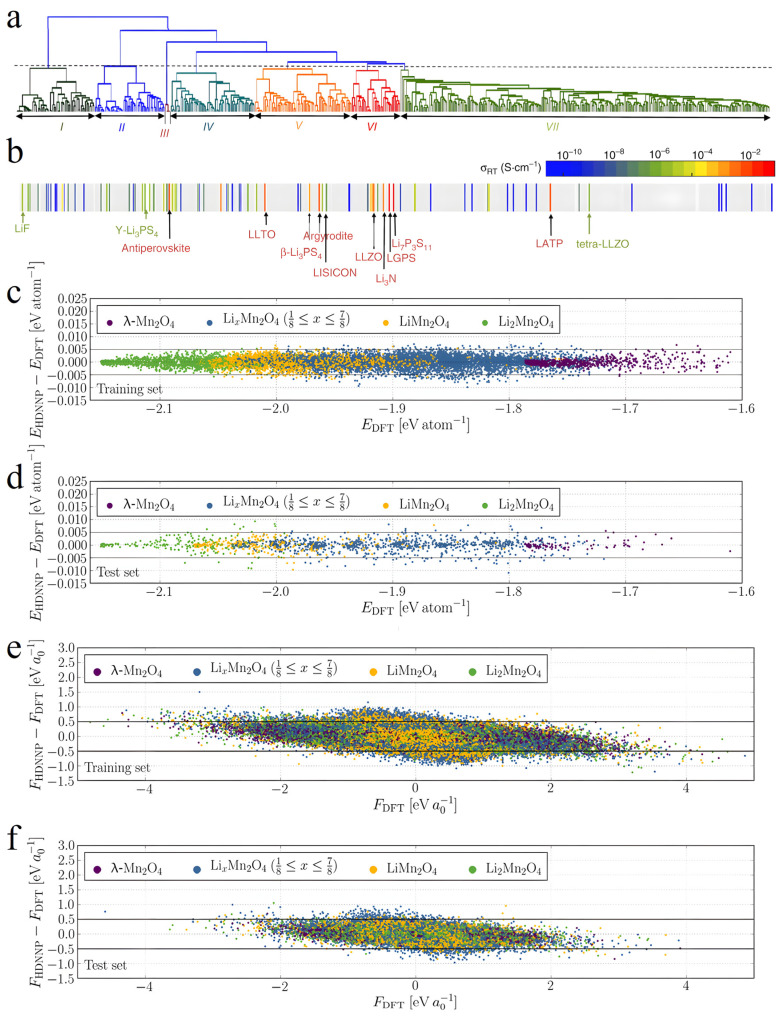
(**a**) Bottom–up tree diagram (dendrogram) generated using the agglomerative hierarchical clustering method. The dashed line shows the position where all compounds are partitioned into seven groups, marked as I–VII from left to right and distinguished by different colors. (**b**) Mapping the dendrogram to the conductivity reveals the grouping of known solid-state Li-ion conductors in Groups V and VI. The color bar shows the scale of σRT. The gray color indicates the conductivity was not measured for the corresponding compound [129]. Signed errors of the formation energies for the (**c**) training set and (**d**) test set and of the force components for the (**e**) training set and (**f**) test set of the HDNNP as a function of the respective DFT values. The data points are colored according to the Li content of the underlying structures. Inside the black lines, the error is smaller than 5 meV atom^−1^ for the energies and 0.5 eV $a_{0}^{-1}$ for the forces, respectively. Reprinted with permission from ref. [130]. Copyright 2020, American Physical Society.

Comprehending the structural attributes of SSE materials and their impact on performance, along with optimizing the preparation procedures, are pivotal for achieving high-performance SSE materials. Leveraging a data-driven approach, AI methods can effectively unveil structure–property relationships and offer insights for optimizing preparation processes. Zhang et al. introduced an innovative approach grounded in unsupervised learning and devised an mXRD-based feature representation technique that converts geometric and topological details of crystal structures into numerical features amenable to ML models [129] (Figure 5a,b). Through Agglomerative Hierarchical Clustering (AHC) and spectral clustering techniques, the team successfully delineated structural similarity patterns in materials, reducing the computational burden of HTS from thousands of compounds to under 100. This methodology not only slashed computational expenses but crucially unveiled the intrinsic link between anionic lattice structures and lithium-ion conductivity.

To explore SSE material phase transitions, Eckhoff’s team harnessed the High-Dimensional Neural Network Potentials (HDNNP) method to scrutinize the structural characteristics of the lithium manganese oxide (Li_x_Mn_2_O_4_) spinel system [130] (Figure 5c–f). By incorporating atom-centered symmetry functions as descriptors, HDNNP effectively captured intricate electronic structural features within the material, encompassing multiple oxidation states and Jahn–Teller distortion. Particularly in the investigation of phase transition dynamics, this approach successfully simulated the orthorhombic-to-cubic phase transition of Li_x_Mn_2_O_4_ for the first time, validated through X-ray diffraction experiments.

**Figure 6:**
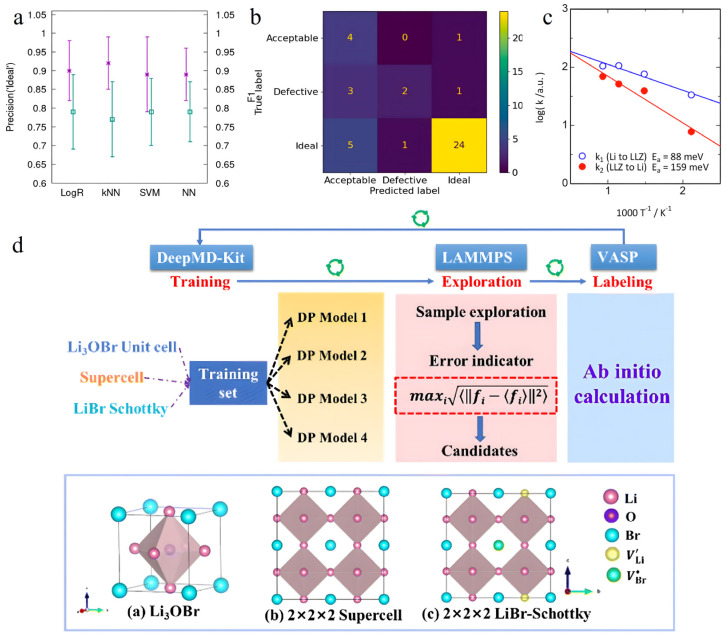
(**a**) Precision of the ideal category (left *y*-axis) represented as stars and F1 (right *y*-axis) represented as squares for the trained multiclassification models on the test set; standard deviation bars are included for both metrics and the precision of the ideal category was used as the reference score. (**b**) Confusion matrix for LogR model whose hyperparameters were tuned using the precision of the ideal category as scoring function [131]. (**c**) Arrhenius plots of rate constants for interfacial Li exchange. The blue line corresponds to the rate constant k_1_ for the reaction from the LLZ phase to the Li phase, and the red line denotes the rate constant k_2_ for the opposite reaction; the slopes of the Arrhenius plots were used to determine the activation energies corresponding to k_1_ and k_2_ (88 and 159 meV, respectively) [132]. (**d**) The workflow of DPGEN and the diagram of training models. Reprinted with permission from ref. [133]. Copyright 2024, The Royal Society of Chemistry.

Gallo-Bueno’s team substantially refined the preparation procedures of composite cathodes for SSBs with polymer electrolyte through integrated ML techniques, particularly supervised learning models [131] (Figure 6a,b). They adopted an SVM model and achieved an impressive R^2^ value of 0.95 in predicting active material loading, while the Logistic Regression (LogR) model attained the accuracy of 0.95 in electrode sample classification tasks. Through Principal Component Analysis (PCA) and feature importance analysis utilizing an RF algorithm, they elucidated the influence of pivotal manufacturing parameters such as viscosity and wet film thickness on electrode performance. Additionally, Maltsev et al.’s research yielded crucial insights for structural optimization [128]. The adopted MLIPs method unveiled that introducing vacancies could notably enhance ionic conductivity when investigating Li_2_B_12_H_12_ material. This discovery aligns with experimental observations where heightened defects from ball milling processes augment ion migration rates, furnishing a theoretical foundation for material structure design and process optimization.

Moreover, Eckhoff’s team proposed an enhanced DL weight initialization method that not only bolstered the training efficiency of HDNNP in multi-hidden-layer neural networks but also proficiently identified absent configurations in material structures by scrutinizing prediction biases across multiple models, offering fresh perspectives for optimizing preparation processes [130].

These studies collectively underscore the pivotal role of AI methods in structural feature analysis and preparation process optimization of SSEs. Through advanced algorithms like DL, intricate structural features of materials and their evolutionary processes can be accurately delineated. Additionally, ML-assisted process optimization enables the efficient determination of key process parameters, guiding the selection of preparation conditions. This data-driven approach furnishes novel research perspectives and tools for the design and preparation of SSE materials.

#### 3.2.3. Interfacial Behavior and Dynamics Simulation

The interfacial dynamics and ion transport kinetics in SSEs play pivotal roles in determining their practical performance. AI methodologies, notably DPMD and neural network potentials, offer novel instruments for investigating these intricate processes. These techniques excel in managing larger-scale interface models and markedly enhance computational efficiency, all the while upholding quantum mechanical precision.

In the exploration of the interface kinetics between the garnet-type SSE Li_7_La_3_Zr_2_O_12_ (LLZ) and lithium metal, Iwasaki’s team achieved notable advancements by employing universal neural network potentials (UNNPs) in conjunction with MD simulations [132]. By constructing a detailed interface model encompassing 1024 atoms, they uncovered a space charge layer, approximately 1 nm thick, near the LLZ/Li interface. Further analysis delved into the energetics of interfacial lithium-ion exchange, revealing an activation energy of 88 meV for transfer from the Li metal phase to the LLZ phase, and 159 meV for the reverse process (Figure 6c). These findings offer crucial microscopic insights into understanding interfacial ion transport mechanisms.

Progress in ion transport kinetics research has also been remarkable. Xia’s team utilized DPMD simulations to investigate lithium-ion diffusion behavior in the Li_3_OBr SSE material, unveiling a linear correlation between defect concentration and ion diffusion capability [133] (Figure 6d). At a LiBr-Schottky defect concentration of 4.2%, the material exhibited an ionic conductivity of 5.71 × 10^−4^ S cm^−1^ at room temperature. Interestingly, the study highlighted that the distribution pattern of defects—whether uniform or random—had negligible impact on ionic conductivity, offering valuable insights for material design.

Employing MLIPs, Maltsev et al. successfully simulated temperature-induced phase transitions and ionic conduction behavior in Li_2_B_12_H_12_ material [128]. Their investigation showcased a superionic state in the disordered β phase, with ionic conductivity reaching 10^−1^ S/cm. Notably, the introduction of vacancies resulted in a substantial enhancement of ionic conductivity in both α and β phases, aligning closely with experimental observations and affirming the reliability of the MLIPs method. Eckhoff’s team leveraged the HDNNP method to comprehensively simulate lithium-ion intercalation, deintercalation processes, and structural phase transitions in Li_x_Mn_2_O_4_ [130]. The distinguishing feature of the HDNNP method lies in its capacity to autonomously identify novel configurations in MD simulations and enhance prediction reliability through multi-model integration. While upholding first-principles calculation accuracy, this method significantly broadens the temporal and spatial scales of simulation, serving as a potent tool for investigating complex interfacial dynamic processes.

These studies underscore the unique advantages of AI methods in probing interfacial behavior and conducting dynamic simulations of SSEs. By capturing intricate dynamic processes that elude traditional methodologies, these innovative approaches not only deepen our comprehension of SSE interfacial behavior and dynamic traits but also furnish robust theoretical underpinnings for the advancement of high-performance SSE materials. With the continual evolution of AI technology, these methods are poised to find broader applications in SSE research, offering enhanced theoretical guidance for the development of ASSBs.

### 3.3. Chapter Summary

This section provides a comprehensive examination of the utilization of AI methodologies in SSE research. By scrutinizing ML and DL techniques, it showcases the distinct benefits of AI technology in predicting materials properties, analyzing structural features, and simulating interface behaviors. In practical scenarios, AI methodologies have exhibited notable efficacy in materials screening, forecasting material properties and identifying potential high-performance candidates [125,126]. Unsupervised learning methods play pivotal roles in unveiling correlations between material structure and properties, as well as refining preparation processes [129,131]. Within the field of interface behavior and dynamic simulations, the adoption of DL techniques has not only bolstered computational efficiency but also enabled extensive, prolonged dynamics simulations [132,133]. Nonetheless, the successful deployment of AI methodologies hinges significantly on the availability of high-caliber training data. This necessitates the seamless integration of traditional theoretical calculation methods with AI technology to harness their complementary strengths. For instance, in high-throughput materials screening, first-principles calculations can be leveraged to generate training datasets, subsequently facilitating efficient predictions through ML models. This synergistic approach, amalgamating theoretical calculations with AI, is extensively explored in the forthcoming chapter.

## 4. Synergistic Application of Theoretical Calculations and Artificial Intelligence

### 4.1. Overview of High-Throughput Materials Screening

High-throughput materials screening as a systematic method for materials discovery and optimization centers on establishing automated screening processes to rapidly identify candidate materials with target properties from vast material spaces. Currently, the initial data for materials screening can be sourced from large-scale computations or public materials databases [134]. Among them, public databases such as ICSD [135], Materials Project [136], and Materials Cloud [137] have accumulated extensive data on material structures and properties, laying an important foundation for HTS. By establishing standardized evaluation processes and screening criteria, researchers can progressively sift through immense pools of candidates to identify potential target materials.

Nevertheless, conventional HTS methods encounter several challenges, particularly when relying on public databases such as Materials Project, AFLOWLIB, and OQMD. These databases frequently exhibit issues of incomplete data, where critical material properties—such as elastic moduli, thermal conductivities, or electrochemical characteristics—are often missing, leaving significant gaps in the datasets. For instance, in Materials Project, while extensive data on crystal structures are available, many materials lack essential descriptors like electronic band gaps or mechanical properties, limiting their utility in comprehensive screening efforts. In addition to data incompleteness, inconsistent data quality is a prevalent problem. Conflicting values for the same material across different databases are not uncommon. For example, discrepancies in lattice parameters or electrode potentials between Materials Project and other computational databases like OQMD can result in unreliable data inputs, leading to inaccuracies in ML models or material predictions. These conflicts arise from variations in computational methods, data recording standards, and experimental conditions. Moreover, multi-source data integration introduces heterogeneity, as datasets collected from experiments, simulations, and literature often differ in format, granularity, and accuracy. In the field of battery research, for example, time-series data used to track electrochemical behaviors frequently exhibit inconsistencies or missing values, such as incomplete records of charge–discharge cycles or irregular timestamps. This heterogeneity complicates efforts to unify data for predictive modeling and reduces the reliability of screening outcomes. Such issues highlight the inherent challenges in using public databases for HTS, as incomplete and inconsistent data undermine the reliability of material screening and discovery processes.

To surmount these limitations, researchers have begun integrating HTS with theoretical calculations and ML techniques. This collaborative approach not only addresses data gaps in databases but also yields profound mechanistic insights through theoretical computations, while expediting the screening process via AI methodologies. This interdisciplinary research strategy presents novel avenues for the exploration and design of SSE materials.

### 4.2. Collaborative Applications

The synergistic fusion of HTS, theoretical calculations, and AI methodologies has emerged as a pivotal strategy for expediting the discovery of new materials and optimizing their performance. This collaborative approach fully leverages the strengths of HTS in large-scale material assessment, the precision of theoretical calculations in characterizing material properties, and the efficiency of ML in data-driven predictions, establishing a systematic research framework spanning from initial screening to performance enhancement. Specifically, the amalgamation of HTS and theoretical calculations facilitates efficient and precise evaluation of material properties. The integration of HTS and ML significantly broadens the scope of exploratory material analysis. Furthermore, the combination of theoretical calculations and ML effectively reduces computational expenses and offers profound mechanistic insights. The collective synergy of these methodologies results in more comprehensive and dependable material design and performance forecasts.

**Figure 7:**
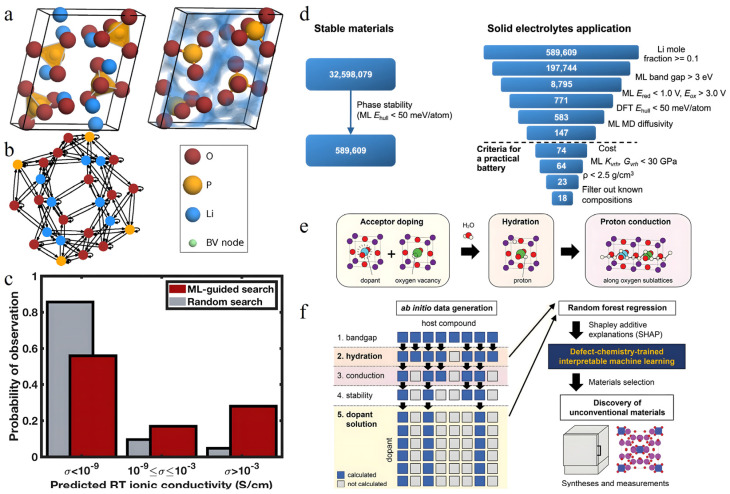
(**a**) Unit cell of Li_4_P_2_O_7_, an example of an SSE candidate. (**b**) Multigraph encoding the Li_4_P_2_O_7_ crystal structure with nodes and edges corresponding to atoms and interatomic interactions, respectively. This multigraph is used as the input to the M3GNet model to predict ionic conductivity from crystal structure. Reprinted with permission from ref. [138]. Copyright 2024, American Chemical Society. (**c**) Normalized histogram of predicted room temperature (RT) conductivities for the ML-guided material search versus the random search. Reprinted with permission from ref. [108]. Copyright 2018, American Chemical Society. (**d**) Main screening stages of the workflow for filtering candidate materials. Of the final 23 candidates, 18 materials have compositions that have not been previously reported. Reprinted with permission from ref. [140]. Copyright 2024, American Chemical Society. (**e**) Activation process for proton conduction in oxides, divided into acceptor doping, hydration, and formation of proton conduction pathway. The introduction of acceptor dopants into the host oxides creates oxygen vacancies and then subsequent hydration incorporates protons into the solid by replacing these vacancies with hydroxyl groups upon exposure to moisture. (**f**) Workflow to explore and discover unconventional proton-conducting oxides, including ab initio data generation for hydration energies and dopant solution energies, construction of interpretable defect-chemistry-trained machine learning models, and proof-of-concept syntheses and measurements [141].

In terms of bilateral collaboration, the combination of HTS and theoretical calculations primarily focuses on improving the accuracy and reliability of material property evaluation. For instance, Xie et al. introduced an HTS strategy that combines bond valence methods with DFT calculations, successfully identifying 329 potential SSE candidates [138] (Figure 7a,b). By utilizing the M3GNet model, accurate predictions of ionic conductivity were achieved through direct feature extraction from material crystal structures. Additionally, BV-KMC simulation, as an efficient theoretical calculation tool, offers a more streamlined approach than traditional DFT calculations for assessing lithium-ion migration behavior. By comprehensively considering pre-screening criteria such as energy above hull (E_H_) and band gap (E_g_), a series of materials with practical application potential were successfully identified.

The integration of theoretical calculations and ML primarily focuses on reducing computational expenses while providing mechanistic insights. For example, Qi et al. developed the LAsou method, which combines ML with active learning algorithms, resulting in nearly a 30-fold efficiency enhancement in predicting stable configurations of Li_10_GeP_2_S_12_ while elucidating the physical underpinnings of configuration stability through theoretical calculations [139]. In another instance, Sendek et al. achieved swift and accurate performance predictions using an ML model trained on atomic structure features, doubling the prediction accuracy compared to conventional methods [108] (Figure 7c). Through integration with DFT/MD, this approach led to the discovery of new superionic conductor materials, including Li_5_B_7_S_13_.

With a three-way collaboration, Chen’s team devised a synergistic predictive system grounded on the M3GNet model framework [140] (Figure 7d). By employing ML models to predict critical properties such as material stability and band gaps, in conjunction with HTS and theoretical calculation validation, they successfully pinpointed 589,609 predicted stable materials from a pool of over 32 million candidates, ultimately unveiling 18 novel SSE materials. Furthermore, Fujii et al. combined high-throughput computational screening with defect chemistry-based ML methods, and through theoretical calculation verification effectively engineered new materials featuring distinctive three-dimensional proton conduction pathways, such as Pb-doped Bi_12_SiO_20_ and Sr-doped Bi_4_Ge_3_O_12_ (Figure 7e,f) [141]. This systematic strategy of “ML prediction–HTS–theoretical verification” not only significantly improved the efficiency of material discovery but also ensured the reliability and feasibility of prediction results.

In conclusion, the collaborative integration of HTS, theoretical calculations, and ML has significantly elevated the efficiency and accuracy of SSE materials research while broadening the landscape of material design possibilities. HTS offers breadth in material evaluation, theoretical calculations guarantee depth in performance forecasting, and ML expedites the entire research process while offering novel design insights. This intelligent research framework presents an effective avenue for swiftly discovering and optimizing high-performance SSE materials, thereby propelling the advancement of ASSB technology.

### 4.3. Chapter Summary

This chapter elaborates on the collaborative applications of HTS, theoretical calculations, and AI methods in SSE research. Through a systematic examination of the integration modes of these three methodologies, their distinct advantages in material discovery and performance enhancement are showcased. In terms of bilateral collaboration, the amalgamation of HTS and theoretical calculations enhances material evaluation accuracy, the fusion of HTS and ML broadens the scope of material exploration, and the convergence of theoretical calculations and ML reduces computational expenses while deepening mechanistic comprehension. The collective synergy of all three approaches yields more comprehensive material design and performance forecasting. Several noteworthy outcomes have been achieved through this collaborative strategy. In material screening, the combination of ML prediction and theoretical calculation validation has led to the successful discovery of several promising new SSE materials. In performance optimization, the collaboration of multiple methods has not only unveiled the intrinsic mechanisms of material performance but also provided clear directions for material design optimization. Particularly in the investigation of complex interfacial behaviors and dynamic processes, this collaborative strategy has demonstrated unique advantages. However, challenges persist in this collaborative approach, including issues related to data quality control, model interpretability, and effective integration among different methodologies. For instance, the reliability of ML models heavily depends on the quality and quantity of input data, which remains a bottleneck due to the limited availability of high-quality experimental datasets. Additionally, the black-box nature of many AI models poses challenges in understanding and validating predictions, which can hinder their adoption in critical applications. Integrating these methodologies effectively also requires the development of standardized workflows and interoperable tools, which are currently in nascent stages. Addressing these challenges necessitates further exploration and refinement in future research endeavors. Overall, with the progression of computational technology and the evolution of AI algorithms, this multi-method collaborative research strategy is poised to assume an increasingly pivotal role in the advancement of SSE materials.

## 5. Conclusions and Prospects

### 5.1. Subsection

This review systematically evaluates the advancements in employing theoretical calculations and AI methods within the realm of SSE research. With the rapid evolution of computational science and AI technology, these methodologies have emerged as indispensable tools for SSE materials research and development, playing pivotal roles in material design, performance prediction, and process optimization.

The realm of theoretical calculations has seen notable progress, with DFT and MD calculations enabling multi-scale characterization of SSE materials. DFT calculations accurately predict electronic structures, band characteristics, and thermodynamic stability, offering theoretical insights for material design. On the other hand, MD simulations provide detailed descriptions of ionic conduction mechanisms and interfacial behaviors, enhancing the understanding of microscopic transport processes within materials. These computational methods establish a robust theoretical framework for elucidating structure–property relationships.

In the domain of AI applications, ML and DL methods have significantly enhanced the efficiency of materials research. By constructing precise structure–property relationship models, these methods swiftly predict material properties and facilitate large-scale material screening. Particularly in material performance optimization and process enhancement, AI methods exhibit distinct advantages over conventional approaches.

The integration of HTS, theoretical calculations, and AI methods in a collaborative manner has given rise to an innovative research paradigm. This synergistic approach not only streamlines material screening processes but also ensures the accuracy of performance predictions, offering systematic solutions for the development of novel SSE materials. Practical implementation has demonstrated that the collaborative utilization of multiple methodologies can expedite the material development process while curbing research and development costs.

### 5.2. Future Development Prospects

Despite the notable progress in theoretical calculations and AI within SSE research, numerous challenges and opportunities lie ahead. Future advancements will primarily concentrate on the following key areas:

First, in terms of computational method development, there is a critical need to enhance the efficiency and accuracy of computational methods, particularly in modeling interfacial reactions and long-term dynamic behaviors. Establishing multi-scale computational models for cross-scale predictions, spanning from the atomic level to macroscopic performance, is imperative. The parallelization and optimization of computational methods will serve as crucial avenues to boost computational efficiency.

Second, breakthroughs in AI technology will center on enhancing model interpretability and expanding application scopes. Developing ML models with increased interpretability will deepen the understanding of factors influencing material performance. The creation of end-to-end material design platforms can facilitate complete process automation from material discovery to performance optimization. Additionally, exploring novel AI algorithms and enhancing model training effectiveness on limited datasets will be key research directions.

Regarding collaborative application innovation, future endeavors will focus on bolstering the seamless integration of experimental data, computational findings, and ML predictions. Establishing standardized material databases and evaluation systems is essential for enhancing data quality and reliability. Moreover, developing intelligent experimental design methods to facilitate efficient integration between theoretical predictions and experimental validation will be a focal point of research.

Expanding application domains will involve actively extending existing methods to investigate new SSE systems and reinforcing applications in interface engineering and process optimization. Notably, exploring the practical utility of these methodologies in the industrialization of ASSBs is crucial, promoting the translation of research achievements into industrial applications.

With the continual enhancement of computational capabilities and the rapid progress of AI technology, theoretical calculations and AI methods are poised to assume an increasingly significant role in SSE research. Through interdisciplinary integration and collaborative methodological innovations, these advanced technologies will significantly accelerate the discovery and development of high-performance SSE materials, thereby propelling the industrialization of ASSBs forward.
